# Infertile men’s needs and assessment of fertility care

**DOI:** 10.1080/03009734.2016.1204393

**Published:** 2016-07-12

**Authors:** Randi Sylvest, Jeanette Krogh Fürbringer, Lone Schmidt, Anja Pinborg

**Affiliations:** a Hvidovre Hospital, Fertility Clinic, Hvidovre, Denmark;; b University of Copenhagen, Department of Public Health, Copenhagen, Denmark

**Keywords:** Family formation, infertility, men, qualitative interview, reproduction

## Abstract

**Introduction:**

Male infertility is potentially a severe, low-control stressor. There is limited knowledge of the expectations, needs, and assessment of fertility care among men with severe infertility. The aim of this study was to explore experience, expectations, needs, and assessment of fertility care among Danish men having severe male-factor infertility.

**Methods:**

Semi-structured qualitative interview study with 10 men with very low sperm quality initiating intracytoplasmic sperm injection (ICSI) treatment at the Fertility Clinic, Copenhagen University Hospital, Hvidovre, Denmark. Five of the men participated in a follow-up interview after their first ICSI treatment. The data collection took place during November 2014 to May 2015. Data were analysed using qualitative content analysis.

**Results:**

Two themes were found: ‘The maze’ and ‘Desire for care’. It felt like an eternity for the men from the referral until treatment started. The men did not understand the process, and it was like being in a maze. The men saw fatherhood as something to strive for. They felt that they could not do what a man is supposed to do, and they felt pushed aside and that treatment focused on the women. The men appreciated the staff’s kindness and professionalism but desired the staff to address emotional subjects too.

**Conclusion:**

The process from referral to treatment felt like a maze for these men. They needed the staff to give them the opportunity to speak of the psychosocial consequences of severe male-factor infertility.

## Introduction

Male infertility is the main or contributing cause in half of the couples who seek fertility treatment (1). It is a widespread international problem as 56% of infertile couples in developed countries seek medical fertility care (2). Men having reduced semen quality frequently want to know why the sperm count is low, and try to find explanations and consequences of this (3).

Infertility concerns both the woman and the man in a couple. The role of the man is often reduced to providing a semen sample on time. This applies even to couples where the male is infertile (4,5). Until now only limited focus has been on men with severe male-factor infertility and their thoughts and experiences regarding their fertility care. A previous study on fertility patients treated at four different public fertility clinics in Denmark (6) found no sex differences in the evaluation of fertility care, except that women were a bit more satisfied than men with how the staff had performed their medical examinations. Both men and women in fertility care in Denmark gave high ratings on medical and patient-centred care.

Male infertility is a potential severe low-control stressor (7), and research has shown that many men with low sperm quality would like to talk about it more widely (3). Mikkelsen et al. found that 72% of 210 Danish men undergoing fertility care lacked information about the psychological consequences of male infertility (8). Approximately 8%–10% of Danish children are born after fertility treatment, and half of these after assisted reproductive technology (ART) treatment.

Usually ‘take-home baby’ rates are used to assess the quality of fertility care, although the importance of patient satisfaction with information provided by the clinic and the perception of the health professionals are increasingly being recognized as indicators of quality of care (9). A previous cohort study showed that fertility patients’ satisfaction with both medical care and patient-centred care was higher when the couple had achieved a pregnancy or delivery and higher with decreasing occupational social class (6). A qualitative interview study among fertility patients showed that they preferred that their treatment took place in a specialized fertility clinic with only a few staff members, a short waiting time, and in a setting where the plan of treatment was known by the doctor and the couple (7,10). Moreover, it has been suggested that a supportive attitude from the health care professionals and the provision of both medical and psychosocial information should be integral aspects of medical care in fertility clinics (5,6,10,11). Finally, there is also evidence that men, unlike women, rarely express their problems and needs in the health service or are more reluctant to do so (12,13).

The aim of this study was to explore experience, expectations, needs, and assessment of fertility care among men with severe male-factor infertility. The study was conducted among men initiating treatment with intracytoplasmic sperm injection (ICSI) in order to optimize the treatment and care of this patient group.

## Material and methods

### Setting

The study was conducted at the public Fertility Clinic at Hvidovre Hospital, University Hospital of Copenhagen, Denmark. All couples initiating ART treatment at our clinic are invited to a scheduled information meeting with the presence of a consultant, laboratory specialist, and a fertility nurse. The meeting lasts for two hours and concerns all aspects of their forthcoming treatment including explanation of the ICSI procedure and indications for ICSI treatment. All men had been referred for specialist andrological examination of their male-factor infertility at an andrological department outside Hvidovre Hospital prior to ICSI treatment in our clinic. This takes in general two to three months and includes ultrasound examination of the testicles, blood hormone and semen analyses, and screening for chromosomal anomalies and Y-chromosome deletions. These investigations take place before the infertile couples can start at our clinic.

Psychosocial support and professional psychosocial services by mental health workers including psychologists are not provided at Danish public fertility clinics. Specially trained nurses and doctors in reproductive medicine provide the fertility care. Psychological counselling in relation to fertility treatment is not mandatory in Denmark. The cost of a maximum of three ART treatments (except medication) is covered by the National Health Service.

### Study population and sampling

Inclusion criteria were men having severe male-factor infertility (≤1 million total motile sperm count (TMC) after processing), planning for ICSI treatment, and having no mutual children. Participants were recruited after they had been informed that the couple needed to undergo ICSI treatment. In that way they had been informed about their very low semen quality. All men were selected consecutively from a log book of treatments at the clinic and from the waiting list. This was done to avoid selection of especially good or bad treatment processes. The participants had different levels of education. Family formation aspirations potentially could be influenced by different levels of education (6). Short-term educational training was defined as vocational training with a year or less of theoretical content, and long-term educational training was defined as four years or more of theoretical training at a university or similar institution. A total of 15 men were contacted by letter and telephone by the researchers (J.K.F. or R.S.). The participants were informed about the aim of the study, that the study included two interviews (one before and one after their first treatment attempt), and that the participants would be anonymous. Four of the invited men did not want to participate, and one of the couples achieved a natural pregnancy before starting ICSI treatment.

Finally, 10 men agreed to participate in the study and were interviewed before their first ICSI treatment, but only half of the participants wanted to participate in the second interview, which took place after their first ICSI treatment. The remaining five did not want to participate because they experienced the treatment as being severely stressful, and some pointed out that they only wanted to be interviewed again when a pregnancy was achieved. The interviews took place between November 2014 and May 2015. They were performed individually to address the emotional aspect of the topic of low sperm quality.

Depending on the participant’s preference, the interview took place in his own home or at the hospital. In total, 10 men were interviewed at the first interview—seven at the fertility clinic, and three in their own homes. At the second interview, five men were interviewed—four at the fertility clinic, and one in his own home.

### Data collection

We developed two semi-structured interview guides. The first interview before treatment initiation focused on family formation intentions, expectations of the ICSI treatment, and thoughts regarding having very low sperm quality. The focus in the interviews was the patients’ expectations of the fertility treatment. The second interview after a first ICSI treatment attempt focused on family formation, wishes and suggestions for information, and how the clinical staff could improve their way of addressing and taking care of severely infertile men’s needs in fertility treatment. Furthermore, very low sperm quality was addressed again. The interview items were constructed in accordance with the experience and knowledge of the researchers and on the basis of previous studies about infertile couples and infertile men (3,4,7,8,10,11).

The interviews were conducted according to Kvale (14) by one of two authors, R.S. or J.K.F. All interviews were performed by only one interviewer. The interviews were audio-taped and transcribed verbatim including non-verbal expressions like silence and laughter, and transcripts were re-checked against the recording by the first authors R.S. and J.K.F. Transcripts were anonymized, and the men were assigned a codename. The duration of the interviews varied from 23 to 80 min with a mean time of 47 min. In general, the atmosphere during the interviews was informal and relaxed.

### Analysis

Transcripts were analysed with qualitative content analysis as used by Graneheim and Lundman (15). The text was analysed with the concepts of meaning units, condensed meaning units, codes, and themes. The analysis was performed in four steps: (1) the interviews were read through to obtain an idea of the content; (2) the text was divided into meaning units, which were defined as words, sentences, or paragraphs in the text, where the content related to each other and to the aim of the study; (3) the meaning units were condensed and labelled with codes, which were abstracted and compared for similarities and differences; (4) the codes were then distributed into themes. Irrelevant text was excluded from analysis. To strengthen trustworthiness, the condensed meaning units, codes, and themes were discussed and reflected on by all the authors throughout the analysis process until agreement was accomplished. Examples of condensed meaning units, codes, and themes are given in Table 1. Quotations were chosen to represent the range of views for each theme.

**Table 1.** Example of analysis; condensation, code and theme.

Condensation	Code	Theme
‘It’s just one big machine … Suddenly, there are just so many people involved in the process.’	Machinery	The maze
‘You stand with a question mark because you can’t get answers to your questions.’	Question mark	The maze
‘You are just a caregiver … It’s not me who is in treatment … You just feel like a caregiver.’	Side-line	Desire for care
‘I feel welcome and comfortable.’	Expectations of the staff	Desire for care

### Ethics

The study followed the principles of the Declaration of Helsinki II for Medical Research. Written informed consent was obtained from all participants. Interviews were anonymized, and sensitive data were kept in a separate document. According to Danish legislation interview studies do not require permission from the Ethical Committee.

## Results

Socio-demographic characteristics and reproductive history about the men are shown in Table 2. Six of the participants had short-term vocational training, and four had long-term vocational training. Of the five who participated in the follow-up interview, there were three with short-term vocational training and two with long-term vocational training.

**Table 2.** Socio-demographic characteristics and reproductive history of the study population.

Alias	Age	Vocational training(short-term orlong-term)	Been withpartner (years)	Time trying toconceive (years)	Male infertilityfactor/mixed maleand female infertility factor	Pregnant afterfirst treatment (yes/no)
1	38	Short	5	2	Mixed	No
2	38	Long	6	2	Male	No
3	34	Long	4	2	Mixed	No
4	40	Short	2	2	Male	No
5	33	Short	6	1	Mixed	No
6	32	Long	5	2	Male	No
7	36	Short	2	2	Male	No
8	40	Short	2	–	Male	No
9	33	Short	3	2	Male	Yes
10	40	Long	4	2	Male	No

We identified two themes: ‘The maze’ and ‘Desire for care’.

### The maze

When the men received information about their very low sperm quality many questions came to their mind along with uncertainty about what comes next, and they lacked information. They felt frustrated because of the fact that they only received written information. They were sent letters and e-mails, but it was one-way communication, and they wanted personal face-to-face meetings and oral information, dialogue, and an opportunity to ask questions. There was a desire for information about the whole process. The men felt frustrated, irritated, and impatient with the process, and they did not understand why the they had to have several sperm tests and other diagnostic procedures.

There was a time period when I didn’t feel like I was doing anything else, other than dropping off semen samples. (Person 1)

It felt like an eternity for the men from the diagnosis time until treatment started. It was like a maze for them, a maze without a map, making it impossible for them to find a path to follow. The staff they met could not give them an overview of the process, of the map. The staff only knew about their own field and not the process for the male patient as a whole. The men wanted to have children now, but it felt like a long ride. Before the men received the information of their very low sperm quality they found themselves at a time in life and at a certain age where it felt right to start a family. The men emphasized that it had to be soon in order to not be too old when they had children. They looked forward to being able to play with their children and being an active part of their lives.

Some men specified that they had found the right woman with whom they wanted to start a family. To make a family of their own would give them another kind of contentment in their lives. When the decision had been made and the desire to start a family was present, they wanted it to happen soon.

… because one wants it so desperately, then one just wishes it would happen tomorrow. (Person 8)

The participants described that they had not always thought about it in this way but that it was something that had occurred with time. In the waiting time before treatment started, they felt like they had been forgotten, and that they were wasting time. They felt like they were in a vacuum, a transfer period, where nothing happened, and they experienced that they did not move ahead.

… We can’t understand at all that it takes such a long time … I’m quite clear that they have to go and take some tests … but four months? … That’s really an unrealistically long time. (Person 9)

The infertile men wanted an overall plan and an overview of the process at the time of the referral. The men expressed that they wanted detailed information about the treatment plan, the results in the process, and information on progress of the treatment.

They wanted a timetable so they could control and manage their lives. The men that had attended the clinic’s information meeting about IVF/ICSI treatment before the first interview were more relaxed and calm compared to the men that had not been to the meeting yet.

A clear overview over the whole process; that’s what I really got out of the information meeting. (Person 2)

One of the men also received good information from the gynaecologist, which meant that he felt much more relaxed compared to the others. He did not feel like he was walking in a maze without a map.

The men were afraid of becoming a number in a big system without personal contact and empathy, and they considered it of great importance that the staff took time in dealing with the couple. They did not want to be treated like they were on an assembly line. The men felt it was like machinery, where a lot of people suddenly got involved in the project of getting a baby.

… It’s just one big machine … Suddenly, there’s just so many people involved in the process. (Person 10)

The men described a family as something good and wonderful, something to strive for. Family was a core value. The surroundings also played a part in the men’s wishes and thoughts about creating a family. They experienced how their acquaintances already had or were starting to have children. The men noticed if other men were fathers. They noticed how the fathers looked after their children and that they had to think of others than themselves. The men had friends that took their children to soccer matches, and that made the men think about their future child’s activities that they looked forward to being a part of. They saw fatherhood as a special and great job in life. They saw it as a huge responsibility, looked forward to it, and had expectations about something amazing waiting for them. Becoming a father was both a dream and something unrivalled, important, and significant to experience.

… the greatest thing. I’d fight for that; it puts a whole new perspective on life. (Person 4)

The private thing of starting a family became a public experience. The privacy and the romantic of getting pregnant and having a child disappeared. They had to say goodbye to the hope of getting pregnant naturally.

… Unromantic fantasy … that one tries for several months, or however long it takes, and then you are happy … I mean, suddenly, 30 people are involved in it. (Person 10)

### Desire for care

Before treatment initiation the men expressed a desire for kindness, understanding, professionalism, and empathy from the fertility clinic staff. They felt it was important that the staff did not look down on them but instead embraced them and held their hands. Furthermore, they wanted to know how the hormones were going to affect their partners.

All the men expressed satisfaction with the information meeting. Here they got the information they needed; statistics and numbers were of particular importance. Before the information meeting many of them sought information through other sources like Google, the clinic’s homepage, the internet in general, or their partners. But after the information meeting they did not have the same need any more, because they had received the information they felt they required.

When the men started treatment, they felt very well informed of the treatment and the plan, and they experienced the staff to be very personal and attentive. The men came to rely on the staff, both in relation to getting an understanding of the situation and getting an overview of the process. They did not feel that they were just a number. The staff were aware of how much it meant to the person sitting in front of them.

Prior to their first treatment the men expressed that they expected involvement in the treatment both as a man and as a couple. They did not wish only to be placed on the side-line. After their first treatment they did, however, consider it natural that the focus was on the women, as it was her body that was being treated and took the biggest strain.

She who gets all the tests, and they’ll need to take some hormones … she’s the one who has the pressure … which I actually think is fine … that the staff are aware that she carries most of the burden. (Person 9)

The men did not consider themselves as emotionally burdened as their partners, and they wished to believe that the ongoing treatment would succeed. They expressed that they did not need psychological help at this point in the treatment. But they did think that maybe it could be relevant with a psychologist for others.

Instead some of the men argued that it would be nice to hear some positive stories of and from former patients.

… one who has been throughout the whole process, someone who could say: ‘Hey; it’s gonna be all right. Of course it’s going to work’. (Person 3)

But there was still a desire for psychosocial care. There was still an inner and emotional confusion. The men wanted to appear strong and supporting for their partners, but it was also a burden for them. They were nervous about whether the treatment would work and help them. Some of them did not feel that the staff were addressing other things like the more emotional aspects of infertility.

It’s not because we talk a lot with the staff. They tell us what is happening, and why it is happening, and what we need to do; but that’s kind of it (as far as information goes). (Person 4)

They tried to push it away, minimize it, and focus on the concrete things and their partner.

… It’s easier to say ‘Hey—I don’t really understand what’s going on’, than it is to say: ‘Hey, I need your help handling some of these feelings that I just don’t understand’ … It’s much more difficult to mention this to them. (Person 2)

Both partners wanted to become parents. They were two persons, a couple, sharing a wish to have a child. The man wanted to shape his part in the treatment himself. He wanted to be active and participate in the things that his partner had to go through. The men wanted to take part in the scanning, the ovum pick-up, and when their partner was going to learn how to inject the medicine.

They wanted the staff to speak to both of them and be attentive towards both of them.

… it was meant for my partner … It wasn’t really that I felt excluded, but it was just a symptom that it was not me who was in treatment … It’d be great if the doctor could just look us both in the eyes. (Person 6)

The men saw it as a couple issue and not as a woman issue.

They knew that they had to put down a semen test at the right time, and additionally they were passive on the side-line. At the same time they emphasized that they were called in from the waiting room with the use of both their names and the fact that there was a chair reserved for them beside their partner during ovum pick-up. The men saw themselves as being expected, important, and allowed to pose questions. The men emphasized being a part of the conversations and the scanning to support their wives, along with the fact that two people hear and remember information better than one.

… it’s important, that we’re both involved in it … since we are both a part of it—it’s not just one of us. (Person 4)

The participants pointed out that it was a treatment that meant a lot to them and their partner.

## Discussion

The findings of the present study were relevant in a broader perspective and internationally as male infertility is a global reproductive health problem (1,2). The major finding was the feeling of being in a maze. We also found that lack of information from the time of referral can lead to impatience, a feeling of being forgotten and left in a never-ending line. Our study showed that information helped the men to see a way out of the maze. Oral and written information about the somatic and psychosocial aspects of infertility and treatment was in high demand, a finding that has been highlighted by others (8). The men wanted face-to-face information about their low semen quality at the time of the referral. The information and time-table helped the men to gain control over their lives (3). Moreover, our results showed that most men wished to support their partner in this process.

When communicating with the couple, the staff should recognize the sense of responsibility the infertile man has towards his partner and try to support his focus on hope and encouragement. The men wished for more than to be at the side-line, but the system placed them there as caregivers though most of them wanted to be involved and included in the process. The present study identified that the men needed the staff’s recognition and acceptance. This finding corroborates previous results that the less the man felt asked about his experience, the less he felt involved as an equal partner (8) and thus highlights that the system needs to see fertility care as a couple issue and not only as a woman issue.

In Denmark the vast majority of men are present at the birth of their children, and furthermore men have the possibility to take paternity leave and parental leave. The man should already during fertility treatment have a well-defined role as an equal partner, which leaves it natural that the fertility clinic’s staff consider both the man and the woman when they meet with the couple (8).

Cues to actions among the participants were that the men found themselves at a time in life when it felt right to start a family with their partner and not being too old for having children. Similar results were found by Eriksson et al. (17), who explored how young adults reflected on future parenthood.

The men noticed if other men were fathers. The surroundings also played a part in the men’s wishes and thoughts about creating a family. The men longed for a time when they could join this community of fathers taking their children to activities, a finding supported also by Tjørnhøj-Thomsen (4).

Our findings confirmed that the men desire kindness, understanding, professionalism, and empathy from the fertility clinic staff. But not all the needs of the men for psychosocial clinical care were met in the clinic. The psychological strains were often unmentioned and unrecognized. In this study the men did not know how to address the emotional aspect of treatment and male infertility. Most men would approach emotional questions by addressing something concrete (13), which was also shown in our study. In working with infertile couples it was important to be aware of the fact that many men did not like the role of being a patient (13). The fact that this was not something which was explicitly addressed by male patients might be due to the tendency in men to be reluctant towards issues relating to illness in general (13). In the present study neither the staff nor the men raised the issue of psychological stress with any frequency. Our study supported previous studies and thus highlighted the need for an informed psychosocial approach to the clinical care of the infertile man (6,10,11,18). Essential to the improved care and counselling of the infertile man was the way the health care system perceives and meets the male patient in open dialogue. This study suggested that infertile men require a greater degree of openness and detail about their condition and its related psychosocial consequences. The data further highlight the fact that the staff were an important source of emotional support since they had frequent contact with the couple. Staff with advanced competence in communication were a sound and effective resource in dealing with the men.

Although many of the men felt that there was a need for more communication about infertility, none expressed the need to be referred to psychosocial counselling at this point. This finding suggested that it is important both to develop ways to help men to engage in counselling and that it is necessary to meet the psychosocial and emotional needs of men. The staff should address that it was normal and acceptable to feel grief in connection with infertility. There was a need for a space where the man’s feelings and needs could be met and accepted. We recommend that more attention should be paid to the provision of comprehensive information about other aspects of reduced sperm quality, including the prior experiences and views of others (e.g. ‘We know from other men …’). With the diagnosis of low sperm count followed an uncertainty and fear that the treatment would not succeed. The wish to hear positive stories could be a way to tackle the emotional confusion, insecurity, uncertainty, and the fear of not succeeding that the men experienced. This supports that it was very important that the staff addressed the psychosocial aspects of the treatment and of having a low sperm count.

We have had the care of men in infertile couples as one of our focus areas in our clinic. One of our goals is that the staff should be aware of men’s desires for care in order to optimize the care of the infertile men. A national leaflet is under development concerning men’s experiences of “being in a maze”, having low semen quality, and starting treatment. As a small adjustment the name of the man is now put on the front page of the clinical file together with the name of the woman to make it easier for the staff to call also the man’s name and not only the woman’s name when the couple are in the waiting room. This is to address that the man is an important part of the couple and takes part in the treatment too.

### Strengths and limitations

In accordance with the standards for qualitative health research suggested by COREQ (19) and Stige et al. (20), analysis and interpretation have been continuously displayed and discussed among the authors and communicated throughout the study. R.S. is experienced in qualitative interviewing specially in family formation. J.K.F. is also experienced in qualitative interviewing and is a fertility nurse with an understanding of reproductive health care which could represent a strength but also a limitation. The men appreciated the fertility nurse expertise, and they took their opportunity to ask questions concerning fertility treatment issues. This implied that there was a need for information prior to treatment and a desire in treatment to talk about psychosocial issues. At the interview before starting ICSI treatment, we accomplished data saturation with 10 interviews. The interviews were quite long, and in the follow-up interview another form of relation occurred between the participant and the interviewer. There was in general a relaxed and open-minded atmosphere in the interview situation. Dependability was created by using the same interview guide. Qualitative studies aim to get a deeper understanding of people’s lived experiences. Striving for generalization was neither possible nor desirable. We think that our results could be transferred to other men with low semen quality in fertility treatment.

A limitation of the present study was that the men in this study might not be representative of all infertile men who undergo ICSI. Men are different, culture is different, and the experience of reduced sperm quality is individual. Furthermore, participation in this study was voluntary, which meant a risk of selection bias. In the follow-up interview only five of the men wanted to participate, and therefore we did not reach data saturation. One of the men mentioned the fact that the treatment was so stressful for both of them and it was harder than expected, so he and his partner needed to pause from the treatment and focus on their relationship. Another stopped halfway through the first course of treatment because of an infection and never started again. Three participants pointed out that they only wanted to be interviewed again when a pregnancy was achieved. This highlights that it is a very emotional subject.

## Conclusion

The process of going from diagnosis to treatment felt like being in a maze, which affected their personal life. The men had reached a point in their lives when they wanted to start their own family. They saw fatherhood as something to strive for, and now they needed help to conceive, and they were impatient. They wanted detailed information about the treatment plan and the opportunity to speak of the psychosocial consequences of their low semen quality. The men wanted information and face-to-face meetings with professionals. They felt like they were placed on the side-line. Fertility care needs to see the treatment as a couple issue and not only as a woman issue. The staff need to focus on involving both the man and the woman in the treatment, and more focus is needed on the men and their psychosocial needs.
